# Inverse association of oxidative balance score with depression and specific depressive symptoms among cancer population: Insights from the NHANES (2005–2020)

**DOI:** 10.1371/journal.pone.0316819

**Published:** 2025-01-17

**Authors:** Hanli Bu, Mingzhen Lv, Linxian Wang, Dan Jiang, Yi Ding, Zheya Wang, Yaoyao Hu, Qin Zhuang, Zhenzhen Tian

**Affiliations:** 1 Department of General Practice, Affiliated Hospital of Jiangsu University, Zhenjiang, Jiangsu, China; 2 Department of Gastrointestinal Surgery, Guilin Medical University Affiliated Hospital, Guilin, China; The University of Sydney School of Medicine, AUSTRALIA

## Abstract

**Background:**

The rising prevalence of depression among cancer patients is alarming. This study examines the relationship between the Oxidative Balance Score (OBS)—a composite measure of dietary and lifestyle factors—and depression, including specific depressive symptoms in this population.

**Methods:**

Data were analyzed from 3,280 adult cancer patients collected in NHANES from 2005–2020. Depression was assessed using the Patient Health Questionnaire-9 (PHQ-9), where a score of 10 or above indicated depression. Symptoms experienced frequently were classified as specific depressive symptoms. Weighted logistic regression models were utilized to explore the correlation between OBS and depression, along with distinctive depressive symptoms.

**Results:**

There was a negative correlation between OBS and depression. The highest quartile of OBS (OR 0.313, 95% CI: 0.161–0.609), along with dietary OBS (OR 0.429, 95% CI: 0.234–0.786) and the third quartile of lifestyle OBS (OR 0.404, 95% CI: 0.226–0.722), was associated with reduced depression risk. OBS was correlated with lower risks of all four somatic depressive symptoms and one cognitive symptom. Dietary OBS was associated with fewer risks of three somatic symptoms and one cognitive symptom. Lifestyle OBS showed a negative correlation with two somatic symptoms. Stratified analyses indicated that the inverse relationship between OBS and depression risk was consistent across subgroups, including females and individuals under 65. a nonlinear association was observed between OBS (p = 0.024), dietary OBS (p<0.001), lifestyle OBS (p = 0.021), and depression.

**Conclusions:**

OBS is inversely related to depression and specific depressive symptoms in cancer patients. Encouraging a diet and lifestyle rich in antioxidants may help reduce the risk of depression in this group.

## 1. Introduction

Depression is a common mental health condition characterized by persistent feelings of sadness, loss of interest or pleasure, changes in appetite or sleep patterns, fatigue, difficulty concentrating, feelings of worthlessness or guilt, and, in severe cases, thoughts of death or suicide [1]. Depression affects approximately 20% cancer patients at a rate about fourfold that of the overall population [2]. However, only around 25% of these patients receive an official diagnosis [3]. It is worth noting that depression substantially reduces cancer patients’ quality of life and potentially results in decreased social interaction, appetite, and sleep, leading to reduced treatment adherence. Furthermore, chronic depressive states may induce physiological changes that affect the growth and spread of cancer. Consequently, depression is linked with increased mortality rates and poorer prognoses in cancer patients [4, 5], underscoring the critical need for addressing this issue within this demographic.

Oxidative stress occurs when there’s an uneven match between the body’s generation of reactive oxygen molecules and its capability to neutralize these harmful compounds or mend the subsequent harm, unsettling the body’s equilibrium, which is critical in both depression and cancer [6]. Studies indicate that individuals with depression exhibit higher signs of oxidative stress and lowered antioxidant levels [7]. This oxidative imbalance may contribute to neuronal damage and compromised neuronal plasticity, leading to the neurostructural and functional changes observed in depression [8]. Furthermore, heightened oxidative stress is linked to DNA damage, gene mutations, disrupted cell signaling, uncontrolled cell proliferation, resistance to cell death, angiogenesis, and the spread of cancer cells, all of which can foster cancer initiation and progression [9]. A multitude of factors can influence levels of oxidative stress, including the consumption of dietary antioxidants like vitamin E and dietary fiber, which play a role in neutralizing free radicals, while dietary fats may exacerbate free radical production [10]. An active lifestyle, coupled with abstaining from smoking and excessive alcohol consumption, may bolster the body’s antioxidant defenses [11, 12]. The oxidative balance score (OBS) was devised to assess the impact of these factors on an individual’s oxidative equilibrium, with a superior OBS indicating a better balance favoring antioxidants over pro-oxidants [13].

Research has shown an reverse between OBS and several health conditions, including depression, diabetes, and other diseases [14, 15]. However, the association between OBS and depression among cancer patients remains unexplored. Moreover, much of the existing research categorizes depression simplistically as either present or absent, neglecting the diversity of depressive symptoms like feelings of sadness, sleep issues and thoughts of suicide, which vary in their biological underpinnings, contributing factors, and consequences [16].

This study aims to delve into the correlation between OBS and depression, including specific depressive symptoms.

## 2. Materials and methods

### 2.1 Study design and population

This study leveraged data from the National Health and Nutrition Examination Survey (NHANES), which utilizes a complex, multistage, probability sampling methodology to achieve a representative sample of the non-institutionalized civilian population in the U.S.

The NHANES received authorization from the Ethical Review Board of the National Center for Health Statistics, with written informed consent obtained from all participants. This study employs publicly available, de-identified data, ensuring compliance with ethical standards and maintaining participant anonymity.

**Fig 1:**
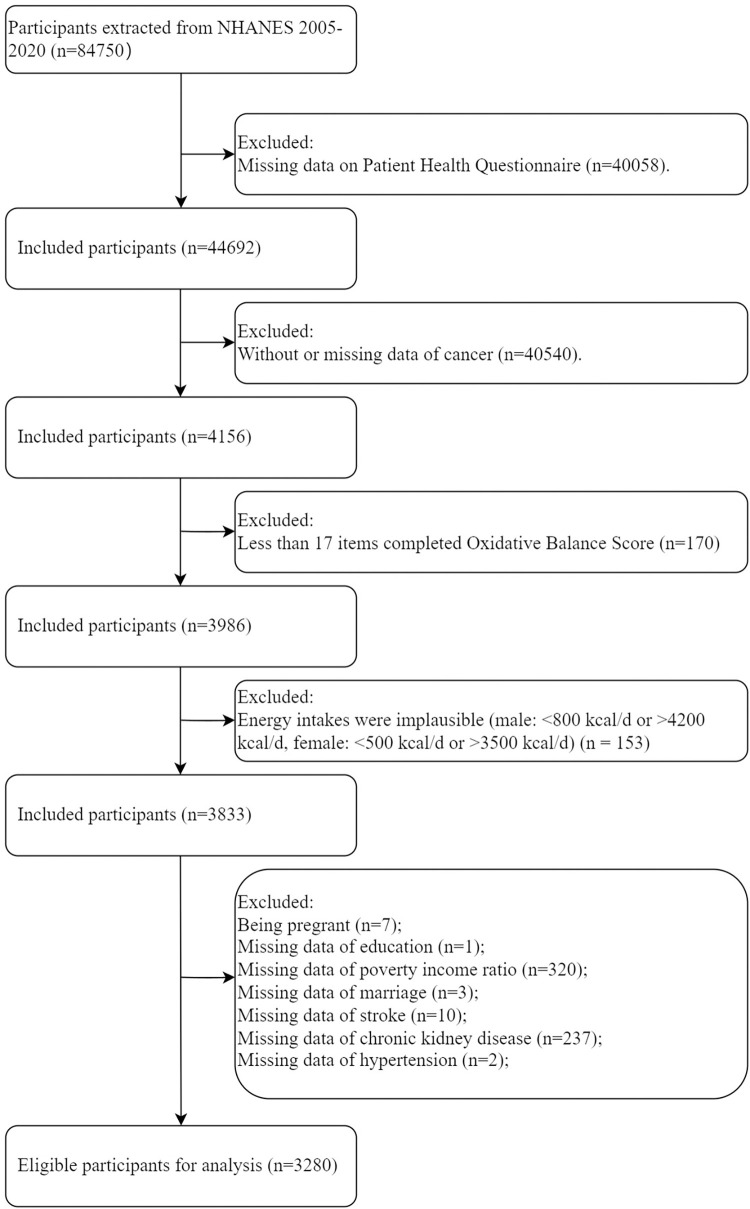
Flowchart of study.

Our analysis focused on individuals who completed the NHANES Questionnaire during the 2005–2020 cycles, encompassing a sample size of 84,750 participants. Exclusion criteria included: (1) absence of data from the Patient Health Questionnaire; (2) lack of cancer-related information; (3) fewer than 17 OBS components; (4) daily energy intake outside the normal range (for males: < 500 kcal/d or > 8000 kcal/d, for females: < 500 kcal/d or > 5000 kcal/d); (5) in a pregnant state; (6) missing data on demographic and health-related factors, such as age, sex, stroke, coronary heart disease, hypertension and so on. It resulted in a final cohort of 3,280 U.S. cancer patients eligible for the study (Fig 1).

### 2.2 Oxidative Balance Score (OBS) evaluation

The OBS was determined by analyzing 16 dietary nutrients and 4 lifestyle elements, incorporating 5 pro-oxidants and 15 antioxidants, based on established research [17]. Information on dietary intake were derived from the first day of diet review interviews, including a wide array of nutrients like carotene, vitamins, minerals, and total fat. Evaluated lifestyle elements comprised physical activity, body mass index (BMI), alcohol intake, and smoking habits, with smoking intensity measured via cotinine levels. Pro-oxidants encompassed total fat, iron, BMI, alcohol intake, and smoking; the remaining elements were deemed antioxidants. Alcohol consumption was categorized into three levels, with corresponding scores [17]: heavy drinkers (women ≥ 15 g/day, men ≥ 30 g/day), non-heavy drinkers (women 0–15 g/day, men 0–30 g/day), and non-drinkers, These elements were further divided into three gender-specific tertiles for scoring, reflecting varying exposure levels. Only participants with data for at least 17 out of the 20 OBS components were analyzed, attributing a score of 0 for any missing data. The study selected participants with data for at least 17 out of the 20 OBS components, assigning a score of 0 for any missing data points. A higher OBS score signified greater antioxidant exposure. The assignment of OBS components is elaborated in S1 Table.

### 2.3 Depression assessment

Depression was assessed using the Patient Health Questionnaire (PHQ-9), gauging the occurrence of depressive symptoms in the last two weeks [18]. Symptoms were categorized into somatic and cognitive symptoms, with respondents rating the severity of each on a four-point scale. Cognitive symptoms of depression include persistent negative thoughts, such as feelings of sadness, worthlessness, hopelessness, difficulty concentrating, impaired decision-making, and suicidal ideation. Somatic symptoms encompassed issues like sleep disturbances, fatigue, appetite changes, and altered motor activity, while cognitive symptoms include the remaining symptoms. Residual symptoms refer to lingering cognitive or somatic impairments, such as difficulty concentrating, low self-esteem or disturbed sleep, that persist after the major depressive episode has subsided.

A cumulative score ranging from 0 to 27 was used to ascertain the presence of depression, setting a threshold score of 10 or higher as indicative of depression. This approach aligns with the methodology of Jokela et al., considering frequent symptom presence for more than half the days or above as a marker of specific depressive symptoms [19].

### 2.4 Covariates assessment

The research took into account several covariates previously linked or speculated to be linked with depression and OBS. These factors included demographic factors, educational background, poverty income ratio (PIR), total dietary energy consumption, and comorbidities such as stroke, chronic kidney disease (CKD), cardiovascular disease (CVD) and others. Methods of diagnosis for these comorbidities were diagnosed through measurements, medication history, and self-report.

### 2.5 Statistical methodology

Data analysis rigorously followed Centers for Disease Control and Prevention (CDC) guidelines, utilizing NHANES data with a unique sample weight derived from the first day’s dietary intake records. OBS components underwent logarithmic transformation for normal distribution approximation. Continuous variables were depicted as weighted means (with standard deviations) or medians (with interquartile ranges), and categorical variables as frequencies (with weighted percentages). Group differences were evaluated using a variety of statistical tests: Rau-Scott chi-squared for categorical variables, weighted univariate linear regression for normally distributed continuous variables, and Kruskal-Wallis for those not normally distributed.

A weighted logistic regression model explored the relationship between OBS, dietary and lifestyle OBS, and depression, as well as specific depressive symptoms in the cancer people, utilizing three models. OBS, dietary and lifestyle OBS were not only treated as continuous variables but were also categorized into quartiles with trend analysis conducted. Model one served as a preliminary model without adjustments for potential confounding variables. Model two incorporated adjustments for age, sex, race, marriage, education, and PIR. Model three extended these adjustments to include dietary energy intake, comorbidities.

Furthermore, this study investigated the relationship between OBS, dietary and lifestyle OBS, and depression within distinct subgroups categorized by age, gender, race, and cancer type. Due to constraints on the size of the cancer participant sample, the analysis was limited to cancer types represented by more than 150 cases. Cancer types with fewer than 150 instances were aggregated under the category "other," as detailed in S2 Table. Weighted restricted cubic splines were employed to explore potential nonlinear relationship. A sensitivity analysis was conducted, which was performed on a subset of 3231 participants with at least 19 out of the 20 OBS components. Statistical analyses were conducted using R4.1.1 with some packages (tidyverse, nhanesR, survey, ggplot2, rms) and significance was determined with two-sided P-values < 0.05.

## 3. Results

### 3.1 Characteristics of cancer participants categorized by depression

Among the 3,280 participants with cancer in this study, 329 were identified with depression. These individuals represent a larger demographic of 18,697,604 Americans diagnosed with cancer, with 1,685,693 suffering from depression. Table 1 presents a comparison of participant characteristics, stratified by depression. Participants suffering from depression were predominantly female, more frequently identified as non-white, unmarried, had lower levels of education, earned lower incomes, and were more likely to have experienced a stroke or chronic kidney disease than those without depression. Participants with cervical cancer had the highest prevalence of depression, with a rate of 19.695%, while participants with prostate cancer exhibited the lowest prevalence at 5.998% (S5 Table)

**Table 1:** Characteristics of cancer participants categorized by depression.

Characteristics	Total	No depression	With depression	P
	(n = 3280)	(n = 2951)	(n = 329)	
Age	65.000(54.000,75.000)	66.000(55.000,75.000)	55.000(46.000,68.000)	<0.001
Sex				0.004
Female	1740(53.049%)	1513(55.555%)	227(68.056%)	
Male	1540(46.951%)	1438(44.445%)	102(31.944%)	
Race				0.003
Non-Hispanic White	2239(68.262%)	2033(86.531%)	206(79.475%)	
Non-Hispanic Black	465(14.177%)	419(5.056%)	46(7.708%)	
Other	576(17.561%)	499(8.412%)	77(12.817%)	
Marriage				<0.001
Single	1321(40.274%)	1133(33.399%)	188(46.446%)	
Couple	1959(59.726%)	1818(66.601%)	141(53.554%)	
Education				<0.001
High school or below	1346(41.037%)	1171(30.816%)	175(46.839%)	
More than high school	1934(58.963%)	1780(69.184%)	154(53.161%)	
PIR				<0.001
≤1.3	754(22.988%)	591(12.245%)	163(36.120%)	
1.3–3.5	1341(40.884%)	1228(35.563%)	113(37.749%)	
>3.5	1185(36.128%)	1132(52.192%)	53(26.131%)	
Energy/kcal	1929.540(19.202)	1930.954(19.831)	1915.272(72.426)	0.835
Stroke				<0.001
No	2989(91.128%)	2716(94.204%)	273(85.793%)	
Yes	291(8.872%)	235(5.796%)	56(14.207%)	
CKD				0.912
No	2134(65.061%)	1917(72.065%)	217(72.474%)	
Yes	1146(34.939%)	1034(27.935%)	112(27.526%)	
CVD				<0.001
No	2483(75.701%)	2276(81.908%)	207(66.659%)	
Yes	797(24.299%)	675(18.092%)	122(33.341%)	
Diabetes				0.311
No	2363(72.043%)	2145(77.084%)	218(73.397%)	
Yes	917(27.957%)	806(22.916%)	111(26.603%)	
Hypertension				0.677
No	1236(37.683%)	1122(42.794%)	114(41.015%)	
Yes	2044(62.317%)	1829(57.206%)	215(58.985%)	
Hyperlipidemia				0.282
No	599(18.262%)	536(17.653%)	63(21.258%)	
Yes	2681(81.738%)	2415(82.347%)	266(78.742%)	

1. The continuous variables were presented as weighted means ± standard deviations or median (P25, P75), and the categorical variables were expressed as unweighted frequencies (weighted percentages)

2. P value was based on Rau-Scott chi-squared test or weighted univariate linear regression or Kruskal-Wallis test where appropriate.

3.PIR: poverty income ratio; CKD, chronic kidney disease; CVD, coronary heart disease

### 3.2 OBS and Its components’ variation in cancer participants categorized by depression

As outlined in Table 2, compared to those without depression, cancer patients with depression exhibited lower overall OBS, dietary and lifestyle OBS levels. Notably, there were significant differences in the log-transformed levels of specific OBS components between the groups (all p<0.05), including dietary fiber, carotene, riboflavin, niacin, vitamin B6, total folate, vitamin B12, vitamin C, vitamin E, calcium, magnesium, zinc, copper, selenium, iron, BMI, and cotinine levels.

**Table 2:** OBS and its components’ variation in cancer participants categorized by depression.

	Total	No depression(n = 2951)	With depression (n = 329)	P
OBS	21.152(0.176)	21.431(0.172)	18.342(0.704)	< 0.001
Dietary OBS	16.700(0.164)	16.907(0.163)	14.604(0.637)	< 0.001
Lifestyle OBS	4.453(0.040)	4.523(0.040)	3.738(0.169)	< 0.001
Dietary OBS components				
Dietary fiber (g/d)	2.635(0.016)	2.656(0.015)	2.427(0.055)	< 0.001
Carotene (RE/d)	4.450(0.040)	4.492(0.041)	4.020(0.147)	0.002
Riboflavin (mg/d)	0.645(0.013)	0.662(0.013)	0.479(0.049)	< 0.001
Niacin (mg/d)	3.009(0.012)	3.020(0.012)	2.891(0.045)	0.007
Vitamin B6 (mg/d)	0.489(0.013)	0.508(0.013)	0.294(0.058)	< 0.001
Total folate (mcg/d)	5.784(0.014)	5.800(0.015)	5.621(0.042)	< 0.001
Vitamin B12 (mcg/d)	1.278(0.019)	1.292(0.020)	1.137(0.068)	0.032
Vitamin C (mg/d)	3.838(0.026)	3.875(0.028)	3.462(0.093)	< 0.001
Vitamin E (ATE) (mg/d)	1.932(0.015)	1.950(0.015)	1.750(0.062)	0.002
Calcium (mg/d)	6.677(0.015)	6.689(0.015)	6.556(0.061)	0.037
Magnesium (mg/d)	5.577(0.011)	5.589(0.010)	5.456(0.045)	0.004
Zinc (mg/d)	2.245(0.014)	2.255(0.014)	2.142(0.053)	0.036
Copper (mg/d)	0.084(0.011)	0.099(0.011)	-0.070(0.045)	< 0.001
Selenium (mcg/d)	4.511(0.013)	4.525(0.012)	4.370(0.051)	0.003
Total fat (g/d)	4.238(0.013)	4.246(0.013)	4.167(0.055)	0.163
Iron (mg/d)	2.525(0.013)	2.540(0.014)	2.375(0.047)	0.001
Lifestyle OBS components				
Physical activity (MET-minute/week)	7.336(0.041)	7.338(0.042)	7.307(0.144)	0.838
Alcohol (g/d)	-Inf (-Inf, -Inf)	-Inf (-Inf, -1.609)	-Inf (-Inf,-Inf)	0.201
Body mass index (kg/m2)	3.350(0.005)	3.344(0.005)	3.412(0.018)	< 0.001
Cotinine (ng/mL)	-3.912(-4.510, -1.715)	-3.963(-4.510, -2.283)	-1.238(-4.135, 5.257)	< 0.001

1.The continuous variables were presented as weighted means ± standard deviations or median (P25, P75)

2.P value was based on weighted univariate linear regression or Kruskal-Wallis test where appropriate.

3.OBS: oxidative balance score; A: antioxidant; P: prooxidant; RE: retinol equivalent; ATE: alpha-tocopherol equivalent; MET: metabolic equivalent.

4.Log transform was performed in all OBS components before comparison.

### 3.3 Relationship between OBS, dietary OBS, lifestyle OBS, and depression in cancer participants

As outlined in Table 3, OBS (OR 0.934, 95%CI: 0.897–0.971), dietary OBS (OR 0.941, 95%CI: 0.905–0.979), and lifestyle OBS (OR 0.798, 95%CI: 0.668–0.955) were inversely related to depression likelihood in numerical analysis. In categorical analyses, the fourth quantile of OBS (OR 0.313, 95% CI: 0.161–0.609) and dietary OBS (OR 0.429, 95%CI: 0.234–0.786), the third quartile of lifestyle OBS (OR 0.404, 95% CI: 0.226–0.722) c significantly lowered depression risk in cancer patients compared to the lowest quartile (all P values < 0.05). These results remained consistent in sensitivity analyses that included more than 19 OBS components (S3 Table).

**Table 3:** Relationship between OBS, dietary OBS, lifestyle OBS, and depression in cancer participants.

	Continuous variable		Classified variable							
	OR 95%CI	P	Q1	Q2 (OR 95%CI)	P	Q3 (OR 95%CI)	P	Q4 (OR 95%CI)	P	P for trend
OBS										
Model 1	0.938(0.910,0.966)	<0.001	1.000	0.525(0.328,0.840)	0.008	0.367(0.221,0.609)	<0.001	0.323(0.200,0.524)	<0.0001	<0.001
Model 2	0.958(0.929,0.989)	0.008	1.000	0.638(0.393,1.036)	0.069	0.478(0.280,0.818)	0.007	0.480(0.287,0.804)	0.006	0.005
Model 3	0.934(0.897,0.971)	<0.001	1.000	0.531(0.316,0.893)	0.018	0.342(0.191,0.614)	<0.001	0.313(0.161,0.609)	<0.001	<0.001
Dietary OBS										
Model 1	0.948(0.920,0.977)	<0.001	1.000	0.566(0.342,0.937)	0.027	0.489(0.281,0.851)	0.012	0.434(0.270,0.699)	<0.001	0.002
Model 2	0.968(0.938,0.998)	0.037	1.000	0.632(0.369,1.084)	0.095	0.643(0.361,1.143)	0.131	0.619(0.375,1.022)	0.061	0.084
Model 3	0.941(0.905,0.979)	0.003	1.000	0.548(0.322,0.934)	0.027	0.460(0.243,0.870)	0.017	0.429(0.234,0.786)	0.007	0.008
Lifestyle OBS										
Model 1	0.727(0.626,0.845)	<0.001	1.000	0.667(0.438,1.015)	0.059	0.314(0.188,0.524)	<0.0001	0.356(0.209,0.608)	<0.001	<0.001
Model 2	0.777(0.663,0.912)	0.002	1.000	0.692(0.449,1.064)	0.093	0.349(0.205,0.594)	<0.001	0.492(0.282,0.857)	0.013	0.002
Model 3	0.798(0.668,0.955)	0.014	1.000	0.716(0.451,1.138)	0.156	0.404(0.226,0.722)	0.003	0.543(0.289,1.019)	0.057	0.020

1.Model 1 was a crude model. Model 2 further adjusted for age, sex, race, marriage, education, poverty-income ratio. Model 3 further adjusted for total energy intake, stroke, cardiovascular disease, chronic kidney disease, diabetes, hypertension, hyperlipidemia.

2.OBS, oxidative balance score; OR, odds ratio; CI, confidence interval

### 3.4 Association between OBS, dietary OBS, lifestyle OBS and specific depressive symptoms in cancer participants

**Fig 2:**
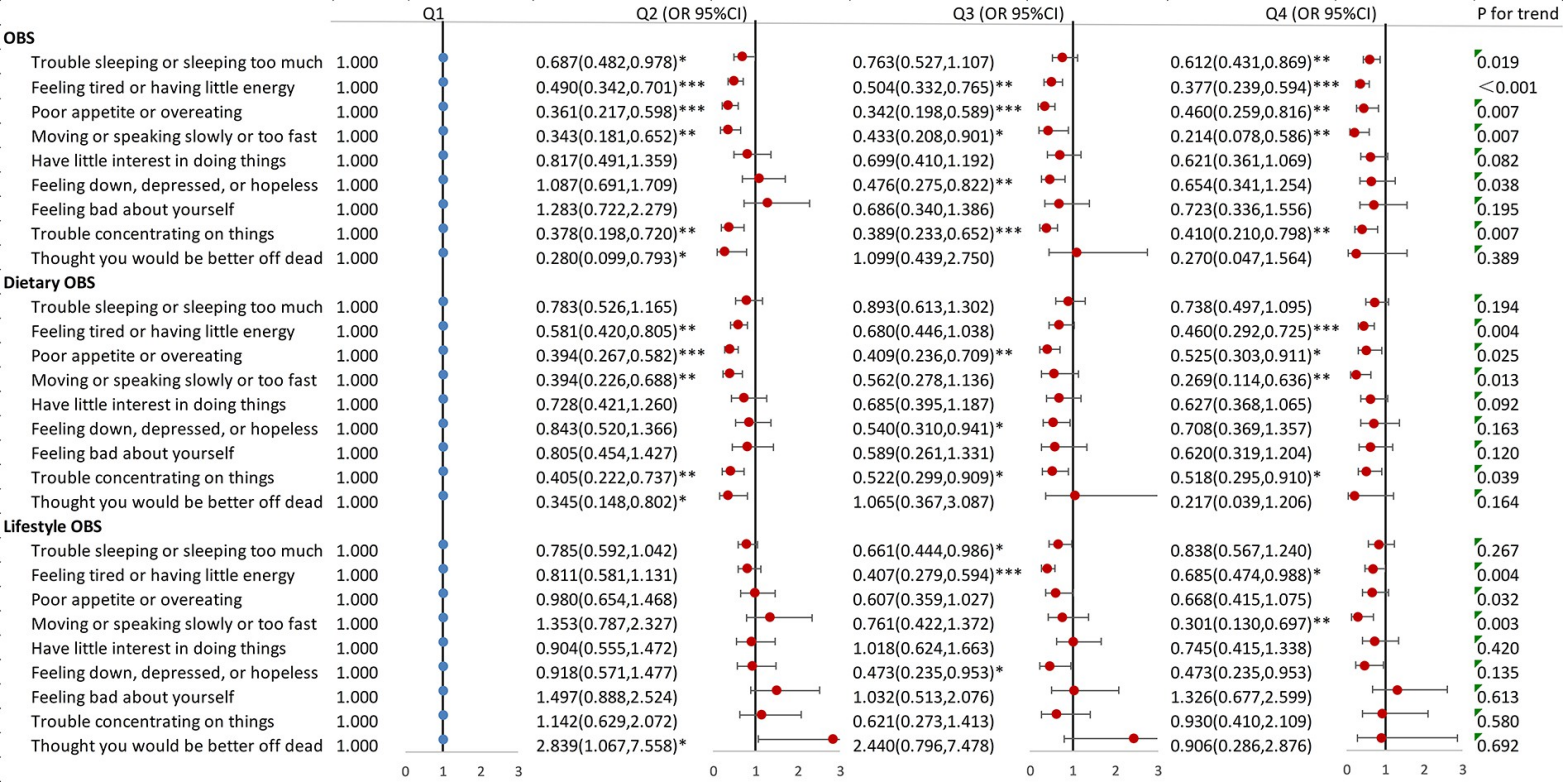
Association between OBS, dietary OBS, lifestyle OBS and specific depressive symptoms in cancer participants. Model adjusted for age, sex, race, marriage, education, PIR, energy intake, stroke, CVD, CKD, diabetes, hypertension, hyperlipidemia.

Fig 2 graphically delineates the link between overall, dietary, and lifestyle OBS and specific depressive symptoms within the cohort of cancer participants. To elaborate, the fourth quartile of OBS reduced the risk for five specific symptoms compared to the first quartile: “Trouble sleeping or sleeping too much” (OR 0.612, 95%CI:0.431–0.869), “Feeling tired or having little energy” (OR 0.377, 95%CI:0.239–0.594), “Poor appetite or overeating” (OR 0.460, 95%CI:0.259–0.816), “Moving or speaking slowly or too fast” (OR 0.214, 95%CI:0.078–0.586), “Trouble concentrating on things”(OR 0.410, 95%CI:0.210–0.798). Similarly, the fourth quartile of dietary OBS was linked with a reduced risk of four specific depressive symptoms: “Feeling tired or having little energy” (OR 0.460, 95%CI:0.292–0.725), “Poor appetite or overeating” (OR 0.525, 95%CI:0.303–0.911), “Moving or speaking slowly or too fast” (OR 0.269, 95%CI:0.114–0.636), “Trouble concentrating on things” (OR 0.518,95%CI:0.295–0.910). Lastly, the fourth quartile of lifestyle OBS was related to a reduced risk of two specific depressive symptoms: “Feeling tired or having little energy” (OR 0.685, 95%CI:0.474–0.988), “Moving or speaking slowly or too fast” (OR 0.301, 95%CI:0.130–0.697). These associations remained stable in sensitivity analyses with over 19 OBS components (S4 Table).

### 3.5 Subgroup analysis and nonlinear relationships

**Fig 3:**
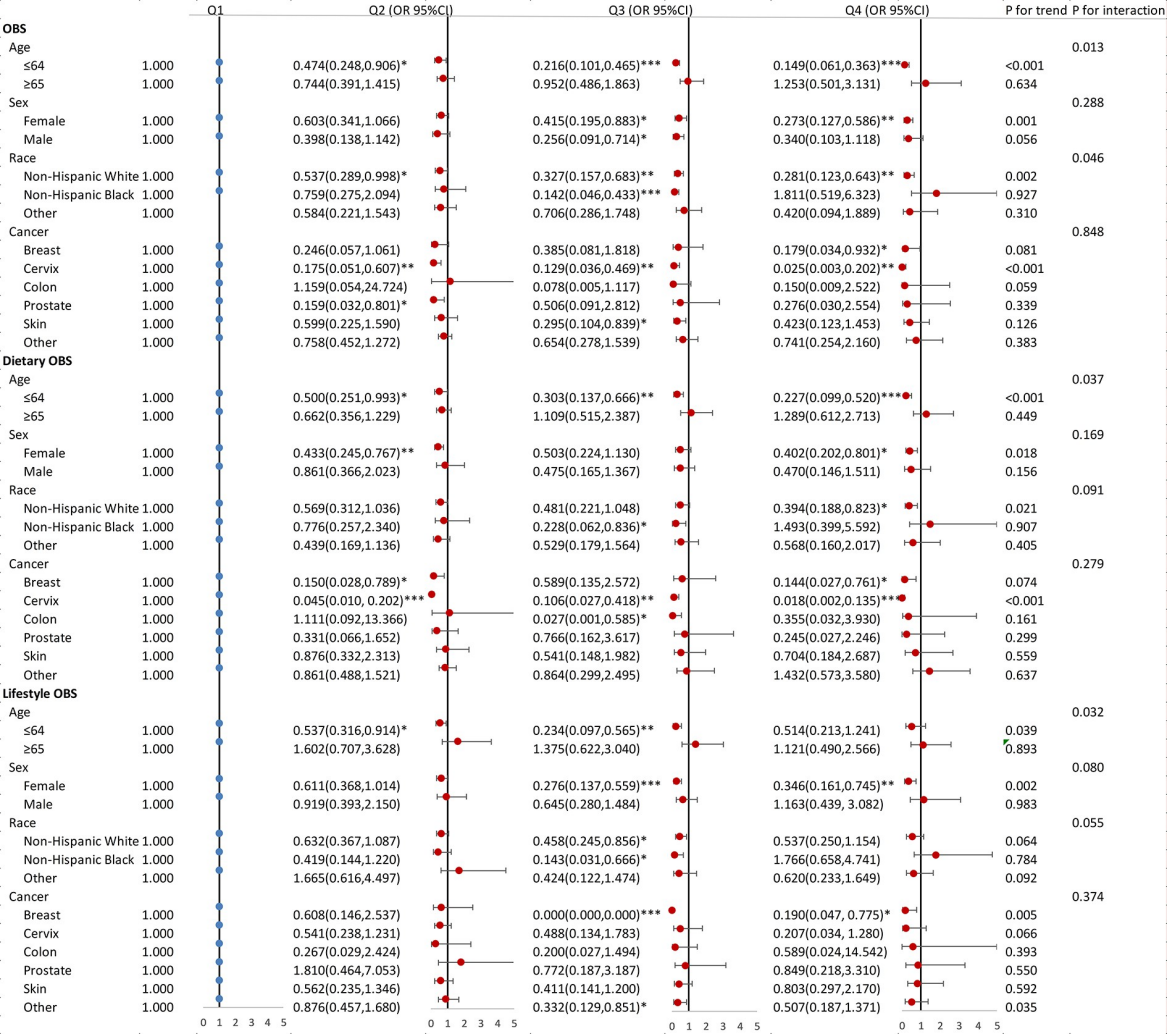
Association between OBS, dietary OBS, lifestyle OBS and depression in cancer participants stratified by age, sex, race, cancer type. Model adjusted for age, sex, race, marriage, education, PIR, energy intake, stroke, CVD, CKD, diabetes, hypertension, hyperlipidemia where appropriate; OBS, oxidative balance score.

Fig 3 demonstrates that OBS and dietary OBS showed a negative correlation with depression risk in participants under 65 years of age (all p-values < 0.05). Significant interactions were detected between age and OBS (p = 0.013), dietary OBS (p = 0.037), and lifestyle OBS (p = 0.032). Among female participants, the highest quartile for OBS (OR 0.273, 95% CI: 0.127–0.586), dietary OBS (OR 0.402, 95% CI: 0.202–0.801), and lifestyle OBS (OR 0.346, 95% CI: 0.161–0.745) significantly reduced depression risk compared to the first quartile, a trend not observed in male participants. OBS and dietary OBS showed a negative association with depression solely among non-Mexican white participants. Furthermore, OBS and lifestyle OBS significantly mitigated depression risk in individuals with cervical cancer, with a similar trend observed among breast cancer patients (all p-values < 0.05).

**Fig 4:**
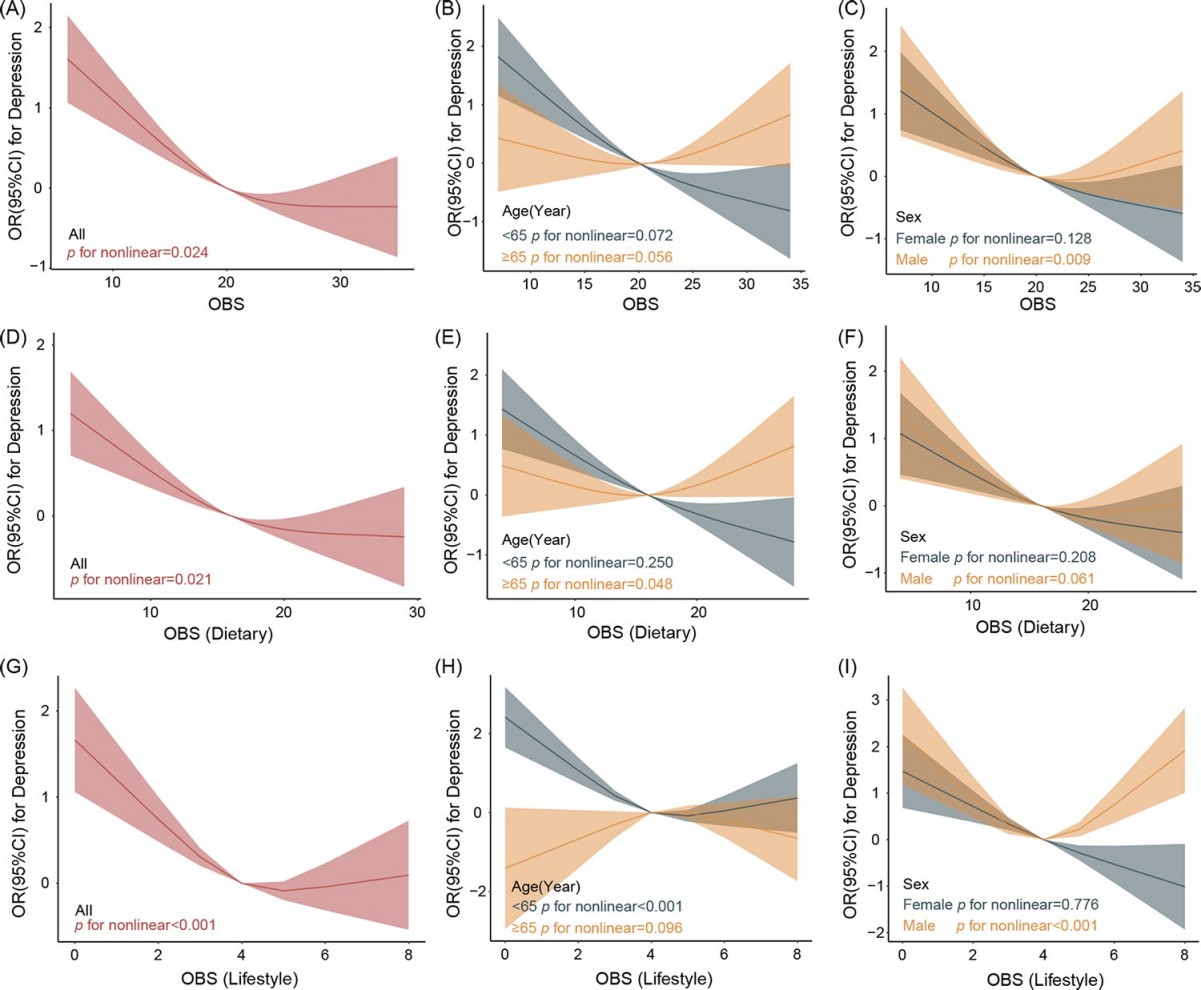
RCS plots of the association between OBS, dietary OBS, lifestyle OBS and depression in cancer participants. Models adjusted for age, sex, race, marriage, education, PIR, energy intake, stroke, CVD, CKD, diabetes, hypertension, hyperlipidemia where appropriate. Solid lines indicate ORs, and shaded part indicate 95% CI.

As is shown in Fig 4, nonlinear inverse relationships were observed between OBS (p = 0.024), dietary OBS (p = 0.021), lifestyle OBS (p<0.001) and depression. Among individuals younger than 65 years, a nonlinear connection was noted specifically between lifestyle OBS and depression(p<0.001). In male participants, nonlinear associations were identified between overall OBS(p = 0.009), lifestyle OBS (p<0.001) and depression.

## 4. Discussion

In this research, we explored the link between OBS and depression, alongside its specific depressive symptoms, within a cohort of adult cancer patients in the United States. The findings indicated an inverse correlation between OBS, dietary and lifestyle OBS and depression. This inverse correlation was predominantly consistent across women, individuals younger than 65 years, non-Mexican whites, and those diagnosed with breast or cervical cancer. Regarding different depressive symptoms, OBS demonstrated a connection with a lower risk of experiencing all evaluated somatic symptoms and one cognitive symptom, dietary OBS was linked to a decreased risk for three somatic symptoms and one cognitive symptom, and lifestyle OBS linked to a decreased likelihood of two somatic symptoms. These results emphasize the importance of adopting diets rich in antioxidants and fostering healthy habits to alleviate depression in cancer patients.

This study represents a pioneering effort in examining the relationship between OBS and depression within the cancer patient demographic. Prior research has mainly concentrated on the impact of OBS or its components on depression in general populations. a study leveraging NHANES data also unveiled negative relationship between OBS, dietary and lifestyle OBS, and depression in adults [14, 20]. Moreover, dietary components such as high fiber intake and essential vitamins have been highlighted for their protective effects against depression [21]. Essential vitamins play a pivotal role in depression. Intakes of dietary β-carotene, vitamin A, and their supplements have been associated with a diminished depression risk [22, 23]. Moreover, depressed individuals generally consume lower quantities of B vitamins, notably among women [24]. A study from Brazil involving 14,737 participants verified an inverse correlation between the dietary intake of selenium, vitamin B6, and depression. Additionally, vitamins C and E, as crucial antioxidants, was lower in depressed patients [25]. Total dietary antioxidant capacity, an indicator of dietary antioxidant capacity, also exhibits an inverse correlation with depression [26]. Antioxidants, such as vitamin E, beta-carotene, and others, can reduce oxidative stress and inflammation by neutralizing free radicals, both of which play a role in the neurobiological mechanisms leading to depression [13]. Lifestyle elements significantly influence depression, with inactivity and heavy drinking heightening depression risk [27]. Physical activity is known to bolster mental health and diminish depression risk, especially in older adults, potentially due to its capacity to mitigate negativity like stress, improve motor function [28]. A high comorbidity exists between alcohol abuse, smoking, and depression [29], with excessive alcohol consumption adversely affecting mood regulation and potentially culminating in depression [12]. Depressed individuals often exhibit higher cotinine levels, regardless of smoking status [30]. A bidirectional relationship exists between BMI and depression, with obesity increasing the risk of depression [31]., whereas depressed patients may gain weight due to medication, reduced physical activity, poor dietary choices, and sleep disturbances. Managing weight can facilitate long-term psychological wellness improvements [32].

Unique to this study is the exploration of OBS’s connection to specific depressive symptoms, revealing a strong link between OBS and all somatic symptoms as measured by the PHQ-9. Although direct evidence connecting OBS to specific depressive symptoms is lacking, certain research groups have identified links between inflammatory responses and depressive symptoms. Oxidative stress can augment inflammatory factors like C-reactive protein (CRP), Interleukin-6 (IL-6) [33]. Inflammatory responses likely serve as intermediaries between oxidative stress and OBS [20]. Another analysis, which also utilized the PHQ-9 for depression assessment, identified that depressive somatic symptoms correlated with elevated levels of inflammatory factors, while depressive cognitive symptoms did not [34]. Additionally, research by Andrea established a significant relationship between high blood CRP levels and physical depressive symptoms in men [35].

Our analysis also delved into the differential impacts of OBS on depression across various subgroups, noting distinct effects based on age, gender, ethnicity, and cancer type. The observed benefits of an antioxidant-rich diet and lifestyle were more evident in patients younger than 65, possibly due to age-related increases in oxidative stress and declines in antioxidant defense mechanisms [17]. Gender differences in the antioxidant capacity, potentially influenced by sex hormones. Estrogen, containing free phenolic hydroxyl groups, possesses antioxidant properties and stimulates the expression and activity of antioxidant enzymes to regulate oxidative stress. On the contrary, androgens can worsen oxidative stress via membrane androgen receptors located on the plasma membrane’s lipid bilayer [36, 37]. Experimental data shows that women generally exhibit a stronger antioxidant capacity compared to men [38]. African Americans are at a heightened risk for excessive oxidative stress [39]. In comparison to Caucasians, African Americans demonstrated higher nitric oxide and IL-6 in umbilical vein endothelial cells and lower levels of superoxide dismutase [40]. Additionally, African Americans exhibited decreased the antioxidant glutathione. Racial disparities in oxidative stress may explain why the protective effects of OBS, dietary OBS, and depression were observed primarily in white Americans within the cancer population. Furthermore, this study unveiled that the protective effects of OBS on depression were restricted to individuals diagnosed with cervical or breast cancer. No such correlations were identified in other cancer types, such as rectal or prostate cancer. Several factors could account for these findings. Firstly, due to sample size limitations, participants with fewer than 150 cases of lung cancer, liver cancer, gastric cancer, and other types were grouped under "other cancers". Secondly, differences in sex hormones contribute to the stronger antioxidant capacity observed in women. Notably, breast and cervical cancer primarily affect women.

This study boasts several advantages. Firstly, individual antioxidant status was evaluated by OBS through modifiable dietary and lifestyle factor. Secondly, this research represents the pioneering endeavor to scrutinize the correlation between OBS and depression, alongside specific depressive symptoms, in cancer populations. Thirdly. the study extensively categorizes individuals by age, gender, ethnicity, and cancer category, enhancing the precision of our findings.

Despite the strengths of our study, several limitations must be acknowledged. First, as a cross-sectional study, our analysis cannot establish causality, highlighting the need for longitudinal or interventional studies to clarify the directionality of the relationship between oxidative balance score (OBS) and depression. Second, the dietary components of OBS were derived from 24-hour dietary recall, which may be subject to recall bias and does not fully capture all oxidative stress-related dietary and lifestyle exposures. Third, depression diagnosis relied on the PHQ-9 questionnaire, widely used in clinical and epidemiological studies, yet unconfirmed by clinical diagnosis. Additionally, depressive symptoms were assessed based on participants’ current psychological state rather than retrospectively at the time of cancer diagnosis. Likewise, the absence of data on cancer stage and treatment, as well as contextual factors like family history of depression, concurrent medication use (including antidepressants) and performance status, limits our ability to explore their potential influence on depression outcomes. It is also important to note that depression and dietary intake were assessed simultaneously after cancer diagnosis, introducing potential bias as both could have been influenced by the diagnosis itself. Furthermore, the PHQ-9 two-week assessment window provides only a snapshot of depressive symptoms and may fail to capture longer-term or recurrent patterns of depression. Lastly, factors related to the COVID-19 pandemic may have slightly inflated depression rates, and the lack of data on physical fitness and tumor staging further limits the scope of our findings. Future studies should address these limitations by incorporating richer clinical details, longer observational or follow-up periods, and broader contextual factors to provide a more comprehensive understanding of the relationship between OBS and depression.

## 5. Conclusion

This investigation reveals an inverse relationship between OBS and depression, as well as specific depressive symptoms, among adult cancer patients in the United States, suggesting that a diet and lifestyle rich in antioxidants may contribute to the prevention and mitigation of depression in this demographic.

## Supporting information

S1 Table Oxidative balance score assignment scheme. OBS: oxidative balance score; A: antioxidant; P: prooxidant; RE: retinol equivalent; ATE: alpha-tocopherol equivalent; MET: metabolic equivalent.
(DOCX)

S2 Table The type and number of cancers of the participants enrolled in study. (DOCX)

S3 Table Weighted logistic regression analysis between OBS, dietary OBS, life OBS and depression in cancer participants(n = 3231). Model 1 was a crude model. Model 2 further adjusted for age, sex, race, marriage, education, poverty-income ratio. Model 3 further adjusted for total energy intake, stroke, cardiovascular disease, chronic kidney disease, diabetes, hypertension, hyperlipidemia; OBS, oxidative balance score; OR, odds ratio; CI, confidence interval.
(DOCX)

S4 Table Association between OBS, dietary OBS, life OBS and specific depressive symptoms in cancer participants(n = 3231). Model adjusted for age, sex, race, marriage, education, poverty-income ratio, energy intake, stroke, cardiovascular disease, chronic kidney disease, diabetes, hypertension, hyperlipidemia where appropriate; OBS, oxidative balance score; OR, odds ratio; CI, confidence interval.
(DOCX)

S5 Table Depression and specific depressive symptoms of participants categorized by type of cancers. The categorical variables were expressed as unweighted frequencies (weighted percentages); P value was based on Rau-Scott chi-squared test.
(DOCX)
