# Supplementary material for: Inverse association of oxidative balance score with depression and specific depressive symptoms among cancer population: Insights from the NHANES (2005–2020)
Source: PLoS One. 2025 Jan 17;20(1):e0316819. doi: 10.1371/journal.pone.0316819 (PMC11741613; doi:10.1371/journal.pone.0316819)
Supplement: S1 Table — OBS: oxidative balance score; A: antioxidant; P: prooxidant; RE: retinol equivalent; ATE: alpha-tocopherol equivalent; MET: metabolic equivalent. (DOCX) [file pone.0316819.s001.docx]

| Supplementary Table 1: Oxidative balance score assignment scheme | | | | | | | | | | |
| --- | --- | --- | --- | --- | --- | --- | --- | --- | --- | --- |
| OBS components | Property |  | Male | |  | |  | | Female |  |
|  |  | 0 | 1 | 2 | | 0 | | 1 | | 2 |
| Dietary OBS components |  |  |  |  | |  | |  | |  |
| Dietary fiber(g/d) | A | ≤12.3 | 12.3-19.8 | >19.8 | | ≤10.1 | | 10.1-16.5 | | >16.5 |
| Carotene (RE/d) | A | ≤42.083 | 42.083-171.417 | >171.417 | | ≤35.861 | | 35.861-155.319 | | >155.319 |
| Riboflavin (mg/d) | A | ≤1.782 | 1.782-2.518 | >2.518 | | ≤1.318 | | 1.318-2.023 | | >2.023 |
| Niacin (mg/d) | A | ≤19.569 | 19.569-28.243 | >28.243 | | ≤14.591 | | 14.591-21.507 | | >21.507 |
| Vitamin B6(mg/d) | A | ≤1.557 | 1.557-2.347 | >2.347 | | ≤1.141 | | 0.076-1.141 | | >1.798 |
| Total folate(mcg/d) | A | ≤286 | 286-457 | >457 | | ≤232 | | 232-350.333 | | >350.333 |
| Vitamin B12((mcg/d) | A | ≤3.14 | 3.14-5.91 | >5.91 | | ≤2.28 | | 2.28-4.38 | | >4.38 |
| Vitamin C(mg/d) | A | ≤35.8 | 35.8-95 | >95 | | ≤28.3 | | 28.3-82.4 | | >28.3 |
| Vitamin E (ATE)(mg/d) | A | ≤5.88 | 5.88-9.83 | >9.83 | | ≤4.747 | | 4.747-7.653 | | >7.653 |
| Calcium(mg/d) | A | ≤664 | 664-1077 | >1077 | | ≤580 | | 580-918.667 | | >580 |
| Magnesium(mg/d) | A | ≤244 | 244-346 | >346 | | ≤199 | | 199-283.333 | | >283.333 |
| Zinc(mg/d) | A | ≤8.89 | 8.89-13.28 | >13.28 | | ≤6.55 | | 6.55-9.92 | | >9.92 |
| Copper(mg/d) | A | ≤0.99 | 0.99-1.407 | >1.407 | | ≤0.798 | | 0.798-1.189 | | >1.189 |
| Selenium(mcg/d) | A | ≤87.1 | 87.1-130.6 | >130.6 | | ≤66.667 | | 66.667-99.233 | | >99.233 |
| Total fat (g/d) | P | >98.81 | 64.37-98.81 | ≤64.37 | | >74.867 | | 50.34-74.867 | | ≤50.34 |
| Iron(mg/d) | P | >17.5 | 11.61-17.5 | ≤11.61 | | >13.34 | | 8.757-13.34 | | ≤8.757 |
|  |  |  |  |  | |  | |  | |  |
| Lifestyle OBS components |  |  |  |  | |  | |  | |  |
|  |  |  |  |  | |  | |  | |  |
| Physical activity (MET-minute/week) | A | ≤960 | 960-3360 | >3360 | | ≤800 | | 800-2446.667 | | >2446.667 |
| Alcohol (g/d) | P | ≥30 | 0-30 | None | | ≥15 | | 0-15 | | None |
| Body mass index(kg/㎡) | P | >30.4 | 26.3-30.4 | ≤26.3 | | >31.573 | | 25.9-31.573 | | ≤25.9 |
| Cotinine(ng/mL) | P | >0.07 | 0.011-0.07 | ≤0.011 | | >0.065 | | 0.011-0.065 | | ≤0.011 |
| OBS: oxidative balance score; A: antioxidant; P: prooxidant; RE: retinol equivalent; ATE: alpha-tocopherol equivalent; MET: metabolic equivalent. | | | | | | | | | | |
|  | | | | | | | | | | |
