# Supplementary material for: Inverse association of oxidative balance score with depression and specific depressive symptoms among cancer population: Insights from the NHANES (2005–2020)
Source: PLoS One. 2025 Jan 17;20(1):e0316819. doi: 10.1371/journal.pone.0316819 (PMC11741613; doi:10.1371/journal.pone.0316819)
Supplement: S2 Table — (DOCX) [file pone.0316819.s002.docx]

| Supplementary Table 2. The type and number of cancers of the participants enrolled in study | | | |
| --- | --- | --- | --- |
| original type | original number | regrouped type | regrouped number |
| Breast | 491 | Breast | 491 |
| Cervix (cervical) | 219 | Cervix (cervical) | 219 |
| Colon | 193 | Colon | 193 |
| Prostate | 495 | Prostate | 495 |
| Melanoma | 213 | Skin | 971 |
| Skin (don't know what kind) | 245 | Skin |  |
| Skin (non-melanoma) | 513 | Skin |  |
| Uterus (uterine) | 133 | Other | 913 |
| Bladder | 76 | Other |  |
| Blood | 8 | Other |  |
| Bone | 13 | Other |  |
| Brain | 14 | Other |  |
| Esophagus (esophageal) | 15 | Other |  |
| Gallbladder | 1 | Other |  |
| Kidney | 66 | Other |  |
| Larynx/ windpipe | 8 | Other |  |
| Leukemia | 28 | Other |  |
| Liver | 10 | Other |  |
| Lung | 67 | Other |  |
| Lymphoma/ Hodgkin's disease | 67 | Other |  |
| Mouth/tongue/lip | 18 | Other |  |
| Other | 156 | Other |  |
| Ovary (ovarian) | 66 | Other |  |
| Pancreas (pancreatic) | 3 | Other |  |
| Rectum (rectal) | 11 | Other |  |
| Soft tissue (muscle or fat) | 3 | Other |  |
| Stomach | 18 | Other |  |
| Testis (testicular) | 17 | Other |  |
| Thyroid | 80 | Other |  |
| TA | 35 | Other |  |

|  |
| --- |
