# Supplementary material for: Inverse association of oxidative balance score with depression and specific depressive symptoms among cancer population: Insights from the NHANES (2005–2020)
Source: PLoS One. 2025 Jan 17;20(1):e0316819. doi: 10.1371/journal.pone.0316819 (PMC11741613; doi:10.1371/journal.pone.0316819)
Supplement: S3 Table — Model 1 was a crude model. Model 2 further adjusted for age, sex, race, marriage, education, poverty-income ratio. Model 3 further adjusted for total energy intake, stroke, cardiovascular disease, chronic kidney disease, diabetes, hypertension, hyperlipidemia; OBS, oxidative balance score; OR, odds ratio; CI, confidence interval. (DOCX) [file pone.0316819.s003.docx]

| Supplementary Table 3. Weighted logistic regression analysis between OBS, dietary OBS, life OBS and depression in cancer participants(n=3231) | | | | | | | | | | |  |
| --- | --- | --- | --- | --- | --- | --- | --- | --- | --- | --- | --- |
|  | Continuous variable |  |  | Classified variable | |  |  |  |  |  |  |
|  | OR 95%CI | P |  | Q1 | Q2 (OR 95%CI) | P | Q3 (OR 95%CI) | P | Q4 (OR 95%CI) | P | P for trend |
| OBS |  |  |  |  |  |  |  |  |  |  |  |
| Model 1 | 0.939(0.911,0.967) | <0.001 |  | 1.000 | 0.562(0.362,0.873) | 0.011 | 0.390(0.243,0.626) | <0.001 | 0.356(0.224,0.566) | <0.001 | <0.001 |
| Model 2 | 0.959(0.930,0.990) | 0.010 |  | 1.000 | 0.697(0.439,1.105) | 0.123 | 0.505(0.307,0.829) | 0.007 | 0.523(0.317,0.864) | 0.012 | 0.007 |
| Model 3 | 0.935(0.898,0.973) | 0.001 |  | 1.000 | 0.602(0.377,0.961) | 0.034 | 0.376(0.220,0.642) | <0.001 | 0.359(0.188,0.684) | 0.002 | <0.001 |
| Dietary OBS |  |  |  |  |  |  |  |  |  |  |  |
| Model 1 | 0.949(0.921,0.978) | <0.001 |  | 1.000 | 0.576(0.346,0.959) | 0.034 | 0.491(0.280,0.863) | 0.014 | 0.445(0.276,0.717) | 0.001 | 0.003 |
| Model 2 | 0.969(0.939,0.999) | 0.045 |  | 1.000 | 0.647(0.377,1.112) | 0.114 | 0.642(0.358,1.154) | 0.138 | 0.630(0.380,1.043) | 0.072 | 0.104 |
| Model 3 | 0.942(0.906,0.981) | 0.004 |  | 1.000 | 0.562(0.330,0.960) | 0.035 | 0.461(0.240,0.885) | 0.020 | 0.440(0.239,0.809) | 0.009 | 0.013 |
| Life OBS |  |  |  |  |  |  |  |  |  |  |  |
| Model 1 | 0.729(0.627,0.848) | <0.001 |  | 1.000 | 0.677(0.443,1.036) | 0.072 | 0.316(0.188,0.531) | <0.001 | 0.360(0.210,0.616) | <0.001 | <0.001 |
| Model 2 | 0.781(0.665,0.916) | 0.003 |  | 1.000 | 0.704(0.456,1.089) | 0.114 | 0.354(0.208,0.605) | <0.001 | 0.498(0.285,0.872) | 0.015 | 0.003 |
| Model 3 | 0.801(0.668,0.959) | 0.016 |  | 1.000 | 0.728(0.456,1.164) | 0.183 | 0.409(0.227,0.737) | 0.003 | 0.548(0.290,1.038) | 0.065 | 0.024 |
| 1.Model 1 was a crude model. Model 2 further adjusted for age, sex, race, marriage, education, poverty-income ratio. Model 3 further adjusted for total energy intake, stroke, cardiovascular disease, chronic kidney disease, diabetes, hypertension, hyperlipidemia. 2.OBS, oxidative balance score; OR, odds ratio; CI, conﬁdence interval; | | | | | | | | | | | |
