# Supplementary material for: Inverse association of oxidative balance score with depression and specific depressive symptoms among cancer population: Insights from the NHANES (2005–2020)
Source: PLoS One. 2025 Jan 17;20(1):e0316819. doi: 10.1371/journal.pone.0316819 (PMC11741613; doi:10.1371/journal.pone.0316819)
Supplement: S4 Table — Model adjusted for age, sex, race, marriage, education, poverty-income ratio, energy intake, stroke, cardiovascular disease, chronic kidney disease, diabetes, hypertension, hyperlipidemia where appropriate; OBS, oxidative balance score; OR, odds ratio; CI, confidence interval. (DOCX) [file pone.0316819.s004.docx]

| Supplementary Table 4. Association between OBS, dietary OBS, life OBS and specific depressive symptoms in cancer participants(n=3231) | | | | | | | | |  |
| --- | --- | --- | --- | --- | --- | --- | --- | --- | --- |
|  | Q1 | Q2 (OR 95%CI) | P | Q3 (OR 95%CI) | P | Q4 (OR 95%CI) | P | P for trend |  |
| OBS |  |  |  |  |  |  |  |  |  |
| Trouble sleeping or sleeping too much | 1.000 | 0.780(0.533,1.143) | 0.201 | 0.846(0.586,1.219) | 0.366 | 0.689(0.483,0.983) | 0.040 | 0.077 |  |
| Feeling tired or having little energy | 1.000 | 0.537(0.389,0.742) | <0.001 | 0.562(0.374,0.844) | 0.006 | 0.414(0.269,0.638) | <0.001 | <0.001 |  |
| Poor appetite or overeating | 1.000 | 0.330(0.204,0.534) | <0.001 | 0.366(0.221,0.605) | <0.001 | 0.494(0.278,0.875) | 0.016 | 0.011 |  |
| Moving or speaking slowly or too fast | 1.000 | 0.407(0.216,0.770) | 0.006 | 0.494(0.240,1.019) | 0.056 | 0.252(0.091,0.696) | 0.008 | 0.017 |  |
| Have little interest in doing things | 1.000 | 0.987(0.584,1.670) | 0.961 | 0.781(0.473,1.291) | 0.332 | 0.654(0.382,1.120) | 0.120 | 0.104 |  |
| Feeling down, depressed, or hopeless | 1.000 | 1.188(0.715,1.974) | 0.504 | 0.485(0.299,0.787) | 0.004 | 0.699(0.368,1.328) | 0.271 | 0.046 |  |
| Feeling bad about yourself | 1.000 | 0.841(0.428,1.653) | 0.612 | 0.522(0.241,1.131) | 0.099 | 0.557(0.247,1.252) | 0.155 | 0.082 |  |
| Trouble concentrating on things | 1.000 | 0.466(0.242,0.900) | 0.023 | 0.457(0.281,0.742) | 0.002 | 0.490(0.255,0.942) | 0.033 | 0.022 |  |
| Thought you would be better off dead | 1.000 | 0.298(0.077, 1.148) | 0.078 | 1.420(0.575, 3.508) | 0.444 | 0.351(0.059, 2.090) | 0.247 | 0.699 |  |
| Dietary OBS |  |  |  |  |  |  |  |  |  |
| Trouble sleeping or sleeping too much | 1.000 | 0.814(0.542,1.223) | 0.318 | 0.936(0.634,1.382) | 0.737 | 0.765(0.509,1.148) | 0.194 | 0.298 |  |
| Feeling tired or having little energy | 1.000 | 0.578(0.413,0.808) | 0.002 | 0.690(0.449,1.060) | 0.090 | 0.456(0.289,0.722) | <0.001 | 0.006 |  |
| Poor appetite or overeating | 1.000 | 0.397(0.268,0.587) | <0.001 | 0.410(0.233,0.722) | 0.002 | 0.542(0.309,0.950) | 0.033 | 0.053 |  |
| Moving or speaking slowly or too fast | 1.000 | 0.402(0.228,0.709) | 0.002 | 0.552(0.263,1.157) | 0.114 | 0.285(0.121,0.674) | 0.005 | 0.025 |  |
| Have little interest in doing things | 1.000 | 0.728(0.419,1.265) | 0.258 | 0.692(0.398,1.201) | 0.188 | 0.582(0.335,1.009) | 0.054 | 0.067 |  |
| Feeling down, depressed, or hopeless | 1.000 | 0.861(0.531,1.397) | 0.542 | 0.544(0.310,0.955) | 0.034 | 0.730(0.382,1.394) | 0.337 | 0.205 |  |
| Feeling bad about yourself | 1.000 | 0.823(0.467,1.453) | 0.499 | 0.560(0.244,1.282) | 0.168 | 0.622(0.321,1.206) | 0.158 | 0.114 |  |
| Trouble concentrating on things | 1.000 | 0.403(0.221,0.735) | 0.003 | 0.519(0.296,0.909) | 0.022 | 0.514(0.292,0.904) | 0.021 | 0.055 |  |
| Thought you would be better off dead | 1.000 | 0.347(0.141, 0.853) | 0.021 | 1.205(0.419, 3.466) | 0.727 | 0.241(0.042, 1.390) | 0.110 | 0.253 |  |
| Lifestyle OBS |  |  |  |  |  |  |  |  |  |
| Trouble sleeping or sleeping too much | 1.000 | 0.832(0.621,1.114) | 0.214 | 0.700(0.472,1.038) | 0.076 | 0.893(0.599,1.331) | 0.574 | 0.431 |  |
| Feeling tired or having little energy | 1.000 | 0.836(0.598,1.169) | 0.292 | 0.420(0.286,0.615) | <0.001 | 0.707(0.486,1.028) | 0.069 | 0.008 |  |
| Poor appetite or overeating | 1.000 | 1.015(0.673,1.530) | 0.943 | 0.625(0.367,1.066) | 0.084 | 0.690(0.427,1.114) | 0.128 | 0.044 |  |
| Moving or speaking slowly or too fast | 1.000 | 1.428(0.831,2.453) | 0.195 | 0.801(0.437,1.470) | 0.471 | 0.316(0.135,0.741) | 0.009 | 0.005 |  |
| Have little interest in doing things | 1.000 | 0.884(0.547,1.429) | 0.612 | 1.028(0.624,1.693) | 0.914 | 0.753(0.418,1.358) | 0.343 | 0.463 |  |
| Feeling down, depressed, or hopeless | 1.000 | 0.929(0.574,1.502) | 0.761 | 0.478(0.237,0.963) | 0.039 | 0.749(0.414,1.352) | 0.334 | 0.145 |  |
| Feeling bad about yourself | 1.000 | 1.537(0.910,2.597) | 0.107 | 1.068(0.528,2.162) | 0.853 | 1.373(0.698,2.700) | 0.355 | 0.548 |  |
| Trouble concentrating on things | 1.000 | 1.127(0.618,2.058) | 0.694 | 0.612(0.267,1.401) | 0.242 | 0.916(0.401,2.092) | 0.833 | 0.558 |  |
| Thought you would be better off dead | 1.000 | 3.147(1.103, 8.974) | 0.032 | 2.693(0.853, 8.505) | 0.091 | 1.025(0.313, 3.361) | 0.967 | 0.592 |  |
| 1.Model adjusted for age, sex, race, marriage, education, poverty-income ratio, energy intake, stroke, cardiovascular disease, chronic kidney disease, diabetes, hypertension, hyperlipidemia where appropriate; 2.OBS, oxidative balance score; OR, odds ratio; CI, conﬁdence interval; | | | | | | | | |  |
|  |  |  |  |  |  |  |  |  |  |
