# Supplementary material for: Inverse association of oxidative balance score with depression and specific depressive symptoms among cancer population: Insights from the NHANES (2005–2020)
Source: PLoS One. 2025 Jan 17;20(1):e0316819. doi: 10.1371/journal.pone.0316819 (PMC11741613; doi:10.1371/journal.pone.0316819)
Supplement: S5 Table — The categorical variables were expressed as unweighted frequencies (weighted percentages); P value was based on Rau-Scott chi-squared test. (DOCX) [file pone.0316819.s005.docx]

| Supplementary Table 5. Depression and Specific Depressive Symptoms of Participants Categorized by Type of Cancer | | | | | | |  |  |
| --- | --- | --- | --- | --- | --- | --- | --- | --- |
| variable | total | Breast | Cervix (cervical) | Colon | Prostate | Skin | Other | P |
| Depression |  |  |  |  |  |  |  | < 0.001 |
| No | 2953(90.988%) | 446(92.834%) | 175(80.305%) | 177(92.005%) | 466(94.002%) | 898(92.517%) | 791(89.275%) |  |
| Yes | 329( 9.012%) | 45( 7.166%) | 44(19.695%) | 16( 7.995%) | 29( 5.998%) | 73( 7.483%) | 122(10.725%) |  |
| Specific depressive symptoms |  |  |  |  |  |  |  |  |
| Trouble sleeping or sleeping too much |  |  |  |  |  |  |  | 0.023 |
| No | 2959(91.762%) | 452(92.709%) | 181(85.783%) | 174(93.690%) | 461(94.011%) | 892(92.941%) | 799(90.231%) |  |
| Yes | 318( 8.140%) | 39( 7.291%) | 38(14.217%) | 17(6.310%) | 33( 5.989%) | 78( 7.059%) | 113( 9.769%) |  |
| Feeling tired or having little energy |  |  |  |  |  |  |  | 0.070 |
| No | 3027(93.970%) | 458(96.087%) | 193(92.161%) | 176(91.803%) | 465(95.067%) | 912(94.850%) | 823(91.883%) |  |
| Yes | 253( 6.016%) | 33(3.913%) | 26(7.839%) | 17(8.197%) | 29(4.933%) | 58(5.150%) | 90(8.117%) |  |
| Poor appetite or overeating |  |  |  |  |  |  |  | 0.003 |
| No | 2674(82.884%) | 408(86.235%) | 155(72.230%) | 153(84.824%) | 428(87.114%) | 820(83.809%) | 710(80.572%) |  |
| Yes | 606(17.100%) | 83(13.765%) | 64(27.770%) | 39(15.176%) | 67(12.886%) | 150(16.191%) | 203(19.428%) |  |
| Moving or speaking slowly or too fast |  |  |  |  |  |  |  | 0.008 |
| No | 2609(80.944%) | 387(81.870%) | 147(72.867%) | 146(69.991%) | 422(84.623%) | 797(82.989%) | 710(80.671%) |  |
| Yes | 669(18.958%) | 104(18.130%) | 72(27.133%) | 47(30.009%) | 73(15.377%) | 170(17.011%) | 203(19.329%) |  |
| Have little interest in doing things |  |  |  |  |  |  |  | < 0.001 |
| No | 2978(91.361%) | 443(91.121%) | 180(83.614%) | 175(94.153%) | 476(95.439%) | 905(93.229%) | 799(88.920%) |  |
| Yes | 302( 8.591%) | 48( 8.879%) | 39(16.386%) | 18( 5.847%) | 19( 4.561%) | 66( 6.771%) | 112(11.080%) |  |
| Feeling down, depressed, or hopeless |  |  |  |  |  |  |  | 0.013 |
| No | 3096(95.039%) | 466(95.643%) | 198(90.190%) | 177(95.638%) | 486(98.159%) | 928(95.810%) | 841(93.769%) |  |
| Yes | 183( 4.909%) | 25(4.357%) | 21(9.810%) | 16(4.362%) | 9(1.841%) | 42(4.190%) | 70(6.231%) |  |
| Feeling bad about yourself |  |  |  |  |  |  |  | 0.071 |
| No | 3061(93.703%) | 462(96.172%) | 195(88.931%) | 177(93.150%) | 474(96.398%) | 916(93.797%) | 837(92.580%) |  |
| Yes | 220( 6.280%) | 29( 3.828%) | 24(11.069%) | 16( 6.850%) | 21( 3.602%) | 54( 6.203%) | 76( 7.420%) |  |
| Trouble concentrating on things |  |  |  |  |  |  |  | 0.207 |
| No | 3142(95.867%) | 469(96.505%) | 203(91.894%) | 182(94.908%) | 481(96.619%) | 940(96.182%) | 867(96.128%) |  |
| Yes | 139( 4.118%) | 21(3.495%) | 16(8.106%) | 11(5.092%) | 14(3.381%) | 31(3.818%) | 46(3.872%) |  |
| Thought you would be better off dead |  |  |  |  |  |  |  | < 0.001 |
| No | 3240(99.039%) | 486(99.560%) | 213(97.898%) | 186(96.559%) | 493(99.047%) | 967(99.893%) | 895(98.436%) |  |
| Yes | 39( 0.906%) | 5(0.440%) | 6(2.102%) | 7(3.441%) | 2(0.953%) | 4(0.107%) | 15(1.564%) |  |
| 1. the categorical variables were expressed as unweighted frequencies (weighted percentages) 2.P value was based on Rau-Scott chi-squared test. | | | | | | | | |
